# Rationale for Using High-Frequency Ultrasound as a Routine Examination in Skin Cancer Surgery: A Practical Approach

**DOI:** 10.3390/jcm13072152

**Published:** 2024-04-08

**Authors:** Diana Crisan, Evelyne Tarnowietzki, Lukas Bernhard, Melina Möller, Karin Scharffetter-Kochanek, Maria Crisan, Lars Alexander Schneider

**Affiliations:** 1 Clinic of Dermatology and Allergology, University Clinic Ulm, 89081 Ulm, Germany; 2 Department of Dermatology, “Iuliu Hatieganu” University of Medicine and Pharmacy, 400347 Cluj-Napoca, Romania

**Keywords:** ultrasound, skin cancer, dermatosurgery, non-melanoma skin cancer, malignant melanoma

## Abstract

Ultrasound and high-frequency ultrasound assessment of melanoma and non-melanoma skin cancer in the pre-therapeutical setting is becoming increasingly popular in the field of dermatosurgery and dermatooncology, as it can provide clinicians with relevant, ”in vivo“ parameters regarding tumor lateral and depth extension as well as potential locoregional spread, cancelling the need of more extensive imaging methods and avoiding a delay in diagnosis. Furthermore, preoperative sonography and color Doppler can aid in orienting the clinical diagnosis, being able in numerous situations to differentiate between benign and malignant lesions, which require a different therapeutic approach. This preoperative knowledge is of paramount importance for planning an individualized treatment regimen. Using sonography at the time of diagnosis, important surgical complications, such as neurovascular damage, can be avoided by performing a preoperative neurovascular mapping. Furthermore, sonography can help reduce the number of surgical steps by identifying the lesions’ extent prior to surgery, but it can also spare unnecessary surgical interventions in cases of locally advanced lesions, which infiltrate the bone or already present with locoregional metastases, which usually require modern radiooncological therapies in accordance to European guidelines. With this review, we intend to summarize the current indications of sonography in the field of skin cancer surgery, which can help us improve the therapeutic attitude toward our patients and enhance patient counseling. In the era of modern systemic radiooncological therapies, sonography can help better select patients who qualify for surgical procedures or require systemic treatments due to tumoral extension.

## 1. Introduction

Skin cancers, mainly comprising melanoma (MM) and non-melanoma skin cancer (NMSC), are the most common malignancies in the white population, showing a continuously increasing incidence rate worldwide [1]. The diagnosis and treatment of these tumors represent a significant problem for health care services worldwide. Treatment options for skin cancers comprise, depending on their extent and type, in some cases, non-surgical methods (e.g., photodynamic therapy, cryotherapy), surgical excision (wide local excision, Mohs, or micrographic controlled surgery), and for locally advanced or metastatic tumors systemic therapy and/or radiotherapy.

Albeit this wide therapeutic portfolio for most skin cancer patients, surgical resection remains the main therapeutic option. The addition of ultrasound (US) and high-frequency ultrasound (HFUS) in the evaluation of skin tumors prior to therapy feeds clinicians and surgeons with significant data regarding the tumor extent, potential infiltration of relevant anatomic structures, or locoregional extent. This information helps the physician with more precise therapeutical planning, similar to a planning CT that aids a radiotherapist in the process of calculating the irradiation field and target volume. Using ultrasound data thus decreases the size of surgical defects and the number of surgical steps, lowers the risk of surgical complications such as postoperative bleeding and/or nerve damage, and spares patients unnecessary operations in cases of locally advanced disease, as identified in the preoperative setting [2,3]. It is also essential when discussing the surgical procedure with the patients, as these should be informed and aware of potential functional neurovascular complications in cases of the vicinity of the tumor to important anatomical structures or the need for cartilage grafting and a secondary surgical grafting site in cases of cartilage infiltration by the tumor [4].

Sonography can also provide clinicians with significant information for differentiating between benign and malignant cutaneous pathology, which has relevant consequences on the therapy and, in some cases, cancels the need for a biopsy or surgery. For skin cancer assessment, color Doppler-equipped ultrasound devices with linear high-frequency probes (>15 MHz), as well as trained operators, are required [5]. In comparison to other imaging devices, such as optical coherence tomography (OCT), confocal microscopy (CM), line field confocal optical computer tomography (LC-OCT), and even computer tomography (CT) or magnetic resonance imaging (MRI), HFUS provides a very good compromise between resolution and penetration depth (up to 60 mm). Thus, it allows us to measure the dimensions of the lesions with high precision. It helps that HFUS has a very good axial spatial resolution and a very accurate definition of the integumentary layers and adjacent structures [6]. The purpose of this review is to illustrate the 10 most common indications for HFUS in skin cancer surgery, which can influence therapeutic management, improve patient counseling and care, and represent a good rationale for employing HFUS as a routine, bedside, cost-effective examination in dermatologic surgery and skin cancer units. The sonographic images depicted in this review were acquired using a Canon Aplio i800 System with a 17LH7 probe.

## 2. Enhancement of Diagnostic Accuracy, Avoidance of Misdiagnosis, Early Identification of Local Relapse

HFUS has proven to be extremely useful in differentiating between benign and malignant lesions and enhancing diagnostic accuracy, omitting preoperative misdiagnosis [2,7].

**Figure 1:**
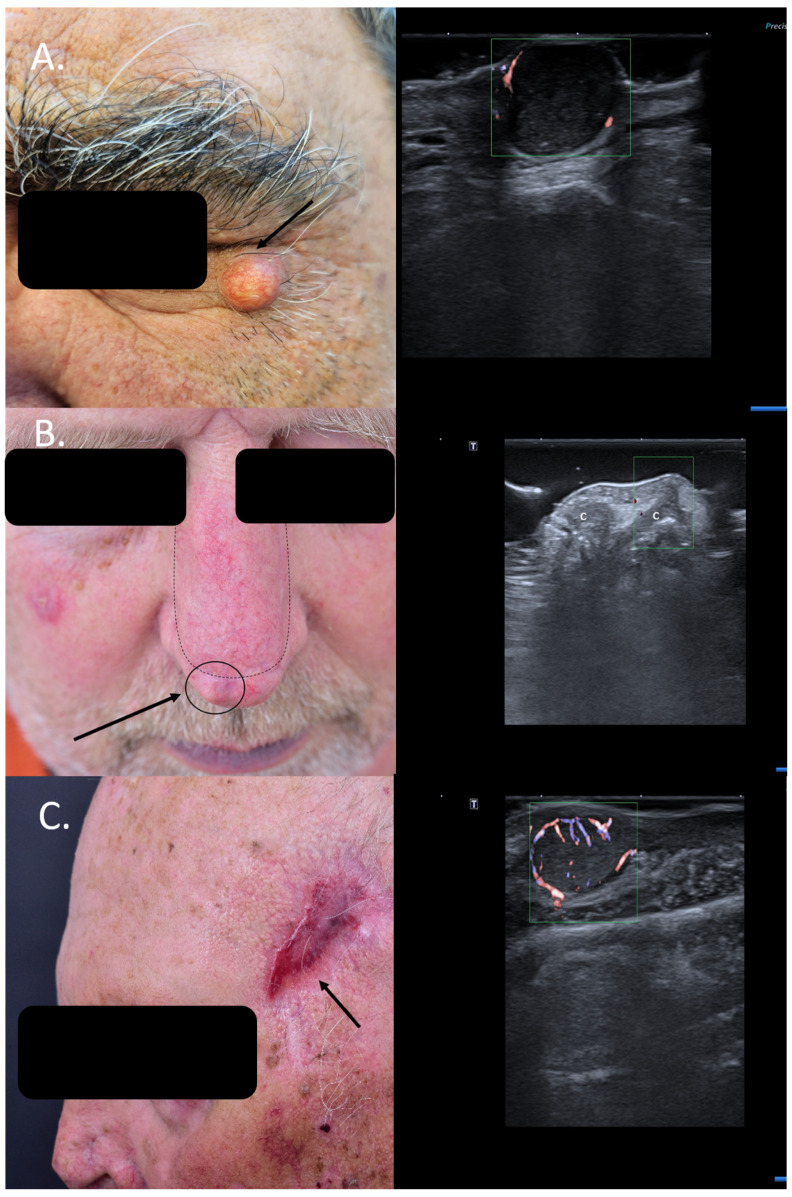
(Clinical aspect—left; HFUS-right): (**A**) Clinical aspect of a solid erythematous nodule with superficial teleangiectasia, as marked by “↑”; HFUS (17 MHz) and color Doppler show a well-defined, dermal-hypodermal, hypoechoic, homogenous lesion with posterior acoustic enhancement and peripheral vascularization, suspicious of a cystic lesion, which was confirmed by histology—transverse view; (**B**) Erythematous nodule, marked by “↑”, next to the surgical scar of a previously resected nasal BCC and reconstructed with an advancement flap, marked by “---”, suspicious of local relapse; HFUS and color Doppler identify a localized cartilage protrusion into the dermis, as marked by “c”, due to the performed advancement flap– transverse view; (**C**) Scar at left temple area after resection of a SCC, as marked by “↑”; HFUS and color Doppler identify next to the scar a hypoechoic dermal-hypodermal, vascularized lesion, suspicious of local satellite metastasis, confirmed by histology—transverse view.

Often, patients are referred to special units with suspicion of skin cancer without histological confirmation, and in many cases, HFUS of the lesion can help to encircle the diagnosis more closely and, in some cases, even spare patients unnecessary surgical interventions. Many cystic lesions at the facial level are easily misdiagnosed as solid basal cell carcinomas (BCCs), as seen in Figure 1A, whereas sonography can, due to specific criteria, immediately differentiate between benign cysts and solid tumors [8].

HFUS can hence help reduce the need for biopsies in sonographic benign-looking cases, decrease perioperative stress and potential functional impairment, and reduce the costs associated with these omitted surgical interventions [9].

Similarly, patients with new bumps within or next to surgical scars of previously resected cutaneous carcinomas are often suspected to have a local relapse. However, this can be easily ruled out or confirmed using means of HFUS and color Doppler. Lesional sonography thus has a significant impact on the therapeutic approach. As seen in Figure 1B, the local nodule lateral to the surgical scar of the advancement flap after resection of a BCC at the nasal level can be clearly identified on ultrasound (US) as a local cartilage protrusion due to the performed flap, canceling the need of re-intervention. In the case displayed in Figure 1C, however, HFUS and color Doppler immediately raise suspicion of a satellite lesion next to the surgical scar of a previously completely resected squamous cell carcinoma (SCC) (6 months prior), which has serious consequences for the case management. Surgical resection confirmed the presence of a satellite metastasis (Figure 1). In such cases, patients require staging due to identified locoregional progress and an interdisciplinary board for discussion of the best therapeutic options Regarding local relapse, studies have shown that the use of HFUS in the follow-up of skin cancer patients following surgery or local therapies can accelerate and also improve the early diagnosis of local recurrence, enabling an early therapeutic intervention [10,11].

Other cases of SCCs initially treated as cysts have also been reported in the literature, where HFUS facilitated the diagnosis and altered the therapeutic approach [12].

**Figure 2:**
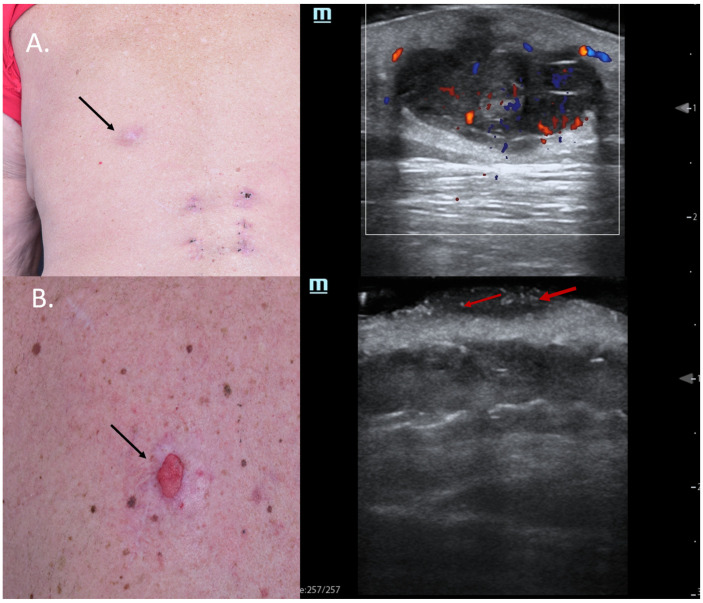
(Clinical aspect—left; HFUS-right): (**A**) Clinical aspect of an ill-defined, erythematous nodule on the left back side, as marked by “↑”; HFUS and color Doppler show a subcutaneous hypoechoic lesion with a central porus connecting the lesion to the surface and central and peripheral vascularization—transverse view; (**B**) Ulcerated plaque on the left back side, as marked by “↑”; HFUS shows a hypoechoic, sharply demarcated lesion of the dermis, with multiple hyperechoic spots, as marked by “↑”, suspicious of an ulcerated BCC, and confirmed by histology—transverse view.

Sometimes, skin tumors may mimic inflammatory lesions such as cystic lesions, and in such situations, color Doppler can help differentiate between the two entities. Figure 2A shows a lesion on the back of a male patient, which was clinically a query epidermal cyst with a central porous, where occasionally greyish material was released. HFUS did indeed show signs of a cystic lesion, with dorsal enhancement and a porous connecting the lesion to the skin surface; however, color Doppler displayed a central and peripheral vascularization suspicious of malignancy. Due to HFUS, the surgical approach was changed from incision and drainage to complete excision, with histology revealing a necrotic amelanotic subcutaneous manifestation of an MM.

Furthermore, in other cases, typical sonographic features might also pinpoint the clinical diagnosis, as seen in Figure 2B. In this case, a patient with MM stage IV with multiple cutaneous metastases under immunotherapy presented with a new, ulcerated lesion suspicious of progressive disease. HFUS clearly reveals a hypoechoic lesion with many hyperechoic spots, suspicious of an ulcerated BCC, which was confirmed using histology (Figure 2).

Hyperechoic spots are typical sonographic findings in BCCs, as described by Wortsman et al., and their presence in higher amounts is associated with recurrence-prone histological subtypes of these tumors [13,14]. Hence, the identification of hyperechoic spots within a lesion by means of HFUS might be very useful in differentiating BCCs from other tumor entities since their presence has not been reported in other skin cancers such as SCC or MM [15]. From a histological point of view, these hyperechoic spots are supposed to represent calcifications, cornified cysts of cellular clusters undergoing parakeratosis or necrosis [13,15,16].

## 3. Lateral Margin Assessment

When dealing with BCCs, the therapeutic choice depends on the histological tumor type (high or low-risk), as well as location, infiltration depth as assessed using biopsy or medical imaging methods, patients’ comorbidities, and preference [17]. However, surgery still remains the mainstay of therapy in BCCs, which is associated with the lowest recurrence rates [18,19].

Especially infiltrative and sclerodermiform BCCs usually show highly irregular margins from a clinical point of view, which makes a clinical border assessment often very difficult. Even though most of these high-risk lesions situated on the face are removed using means of micrographic controlled or Mohs surgery, pre-surgical sonographic scans may help surgeons improve tumor margin determination and hence reduce the number of surgical steps required for achieving complete resection. It can also prevent the removal of excessive tissue in highly aesthetic areas, which often leads to large surgical defects, which require complex reconstructions and can lead to significant scarring in the face.

**Figure 3:**
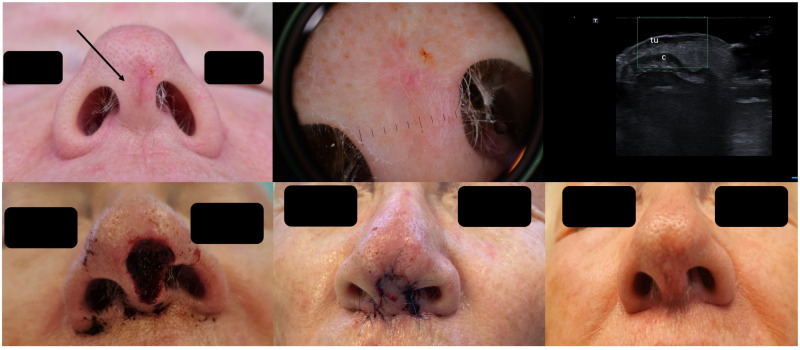
(Clinical, dermoscopic, and HFUS aspect—upper image; surgical approach—lower image): Clinical aspect showing an irregular, unsharply demarcated macule at columella level, as marked by “↑”, consistent with a BCC; Dermoscopy shows irregular dot and arborizing vessels; HFUS and color Doppler show a hypoechoic, non-vascularized lesion of the columella (“tu”) with no cartilage infiltration, as marked by “c”; Clinical aspect after complete tumor resection, defect closure with an advancement flap from the columella and clinical aspect at 8 weeks follow-up.

As seen in Figure 3, the clinical presentation of the BCC at the columella level leaves one with insecurity regarding the lateral tumor extent; dermoscopy does not help any further; however, HFUS can clearly identify the lesion’s subclinical lateral extension, enabling a complete tumor resection in one surgical step as confirmed using histology and reconstruction with an advancement flap [20] Figure 3.

While older and larger ultrasound transducers showed clear limitations in assessing tumor margins in certain facial areas such as the nose, ears, and eyelids (known high-risk sites for BCCs), modern small hockey-stick-like transducers facilitate a very precise tumor mapping even in irregular lesions, which is essential for the estimation of tumoral extension and surgical planning, including the identification of optimal techniques for surgical closure [19].

## 4. Assessment of the Tumor Infiltration Depth

The tumor infiltration depth cannot be obtained using means of clinical examination only, and often, tumor biopsies are rather superficial, where the complete tumor infiltration depth is not represented. This critical piece of information is, however, essential for surgical planning in skin cancer, and sonography has the advantage of being able to assess the tumor infiltration depth very accurately in comparison to dermoscopy, CM, and OCT, which present relevant penetration issues, being limited by their superficial penetration <2 mm [4,21,22].

**Figure 4:**
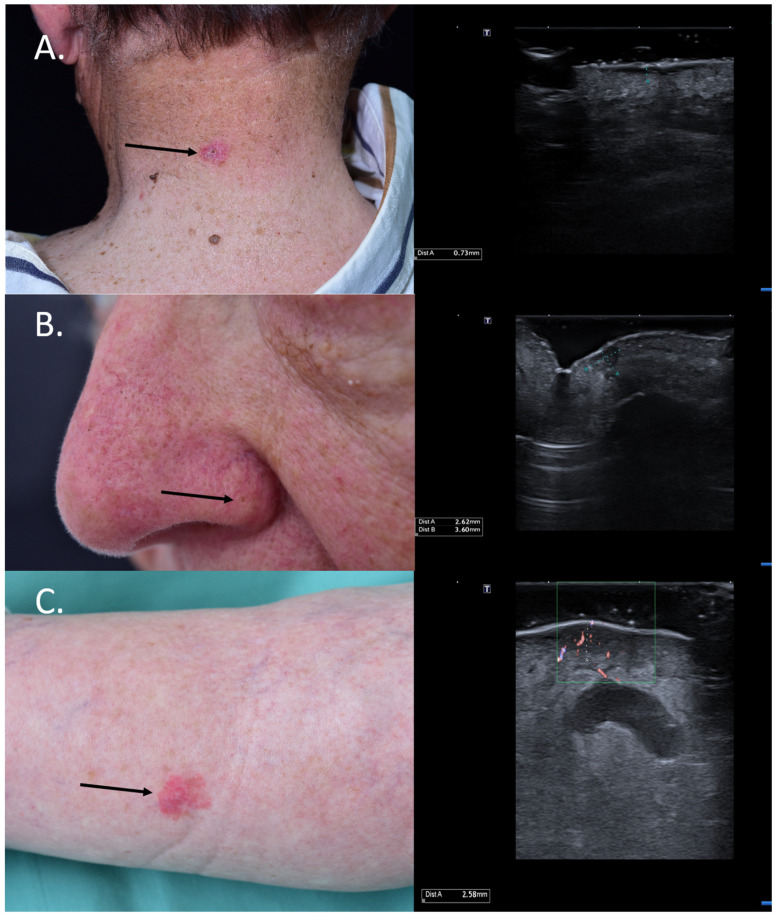
(Clinical aspect—left; HFUS-right): (**A**) Clinical aspect showing an erythematous plaque of the neck, as marked by “↑”, consistent with a superficial BCC; HFUS showing a superficial, spindle-shaped lesion with a tumor infiltration depth of 0.7 mm—transverse view; (**B**) Clinical aspect showing an erythematous macule on the left nasal ala, as marked by “↑”, consistent with a BCC; HFUS identifies a hypoechoic lesion with hyperechoic spots within and a tumor infiltration depth of 2.6 mm—longitudinal view; (**C**) Erythematous patch of the left lower leg, consistent with a BCC, as marked by “↑”,; HFUS and microvascular imaging display a hypoechoic, ill-defined dermal-hypodermal lesion with basal vascularization and a tumor infiltration depth of 2.5 mm; underneath the lesion, section thru the great saphenous vein—transverse view.

For BCCs, the knowledge of the tumor infiltration depth is essential in choosing between a surgical and non-surgical treatment, as superficial and low-risk BCCs < 2 mm can also benefit from non-surgical methods such as photodynamic therapy (PDT) [23]. Figure 4 HFUS was also shown to be extremely valuable in differentiating between actinic keratosis (AK) and invasive SCC by assessing the layer involvement, which would allow clinicians to noninvasively monitor AK lesions and intervene using means of surgery in case of invasiveness and suspicion of SCC [24]. Furthermore, HFUS in the preoperative setting can identify high-risk SCCs by measuring a tumor depth of >6 mm, which need a closer follow-up. Such tumors have a higher risk of recurrence and metastasis [25]. Lastly, for MM, studies have shown that the sonographic infiltration depth correlates very well with the histological Breslow index; the knowledge of this information in the preoperative setting has significant implications for the decision to perform a sentinel node biopsy and the required safety margins, which have to be considered [26,27,28,29].

## 5. Tumor Mapping and Evaluation of Operability

US and HFUS have been proven to be extremely effective in identifying a bone invasion by skin tumors, canceling the need for other medical imaging methods, such as CT, which is not always available and requires contrast agents (often unfeasible in elderly patients with renal insufficiency) and usually delays the diagnosis [30]. In cases where HFUS identifies a localized bone infiltration, surgery still remains a therapeutic option, and HFUS can help guide the surgery by identifying the precise infiltrated area that needs to be resected. Nonetheless, these procedures require general anesthesia, sometimes an interdisciplinary team, and patients can often develop significant postoperative complications.

**Figure 5:**
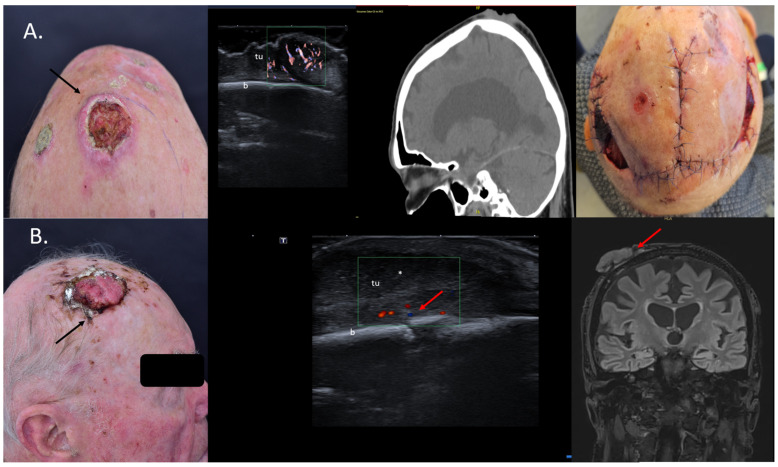
(Clinical aspect—left; HFUS, CT, MRI—middle; postoperative aspect—right): (**A**) Clinical aspect of an exulcerated tumor at the parietal area, as marked by “↑”, consistent with an SCC; HFUS and microvascular imaging showing an irregular hypoechoic, highly vascularized lesion (“tu”) extending up to the bony surface (“b”) with no bone infiltration—transverse view; CT scan showing no bone infiltration by the tumor mass; Clinical aspect after resection and partial defect closure with two rotation flaps; (**B**) Clinical aspect of an exulcerated SCC of the right parietal area, as marked by “↑”; HFUS and color Doppler display a hypoechoic lesion with suspicion of bone infiltration, as marked by “↑”—transverse view; MRI showing local bone infiltration, as marked by “↑”—coronal view.

In the dermatosurgical setting, we are often confronted with large tumors, especially SCCs situated on the bold scalp, where sonography can help to map the lesions with high precision and rule out or confirm a bone infiltration, as seen in Figure 5. For such cases, there is no need for additional CT or MRI, as sonography is superior for the evaluation of soft-tissue lesions due to its high resolution [31], see Figure 5.

According to Wortsman et al., US is the only imaging method that can define the tumor infiltration depth without any penetration issues, as well as the best method to characterize the tumor in all axes while also performing a locoregional staging [22]. Cases with bone involvement are usually discussed in interdisciplinary tumor boards in order to present the patients with the best therapeutic options: surgery, systemic treatment, and/or radiation therapy [32].

## 6. Identification of Cases for Mohs Surgery

Mohs or micrographic controlled surgery is usually a preferred surgical option in cases of skin cancer, mostly BCCs and SCCs located in areas where as little as possible tissue should be removed, such as the face (nose, eyelids, ears, lips), scalp, nails, genital region, etc. It is also preferred in skin cancer cases associated with high relapse risk, such as sclerodermiform BCCs, for instance, as assessed by histology or in cases with perineural infiltration or relapsing lesions [33]. As evaluated by Wortsman et al. [22], the presence of ≥7 hyperechoic spots within BCC lesions on HFUS was associated with recurrence-prone histological subtypes, such as micronodular, morpheaform, and sclerosing BCCs, enabling the use of HFUS for the identification of high-risk tumors, which would benefit from Mohs surgery. In this retrospective study, a sensitivity of 79% and specificity of 53% were seen to predict the high risk of recurrence subtypes [13].

**Figure 6:**
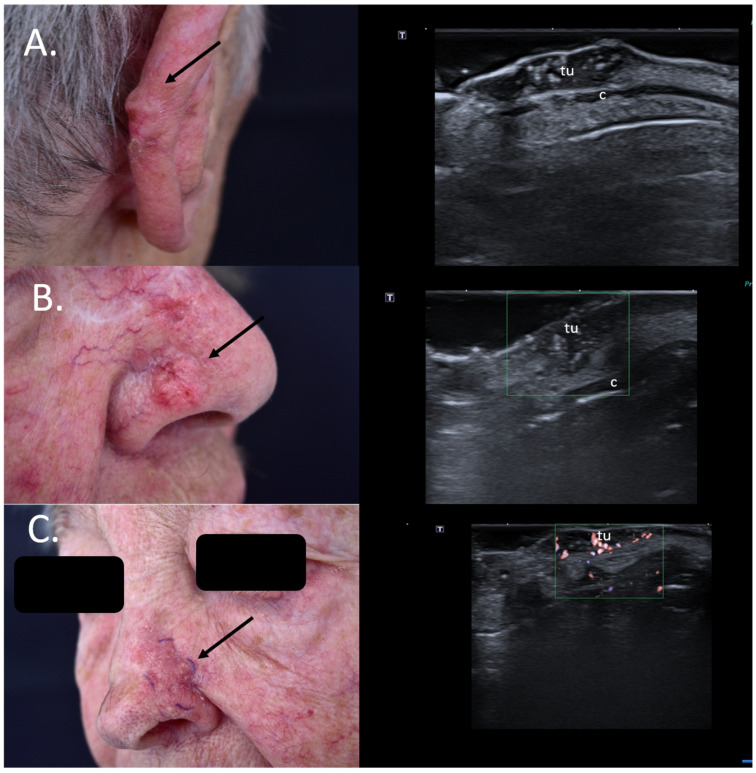
(Clinical aspect—left; HFUS—right): (**A**) Clinical aspect of an erythematous nodule with arborizing vessels, as marked by “↑”, consistent with a BCC; HFUS showing a hypoechoic lesion and numerous hyperechoic spots within, with no contact to the cartilage, as marked by “c”—longitudinal view; (**B**) Erythematous nodule on the right nasal wall, as marked by “↑”, consistent with a solid BCC; HFUS displaying a hypoechoic, lesion protruding into the dermis with multiple hyperechoic spots and no cartilage infiltration, as marked by “c”- transversal view; (**C**) Surgical scar at left nasal ala after surgical resection of a BCC, as marked by “↑”; HFUS und microvascular imaging showing a hypoechoic, vascularized lesion beneath the surgical scar, consistent with local relapse, as marked by “tu” – transversal view.

As seen in Figure 6, the upper two lesions presenting many hyperechoic spots within the tumor were both infiltrative subtypes on histology and, hence, high-risk lesions, which should be removed using micrographic controlled surgery not only due to their location but also due to the histological subtype associated to a high relapse risk Figure 6.

Additionally, for relapsing BCCs, the preoperative identification of the tumoral extent below the surgical scar provides surgeons with significant information for adequate surgical planning [34] Figure 6.

## 7. Choice of Surgical Approach

When dealing with skin cancer in highly aesthetic areas such as the nose or auricles, identifying a potential cartilage infiltration by the tumor mass is very important for surgical management and reconstruction choice [28]. Firstly, the preoperative detection of cartilage infiltration by the tumor mass can enable surgeons to reduce the number of surgical or Mohs steps by directly resecting the infiltrated cartilage; further, this knowledge is of great use for planning the surgical reconstruction, which in some cases requires a more extensive resection and cartilage transplantation from the auricle or even ribs, leading to a secondary surgical site, situation which has to be discussed with the patient in advance [4].

**Figure 7:**
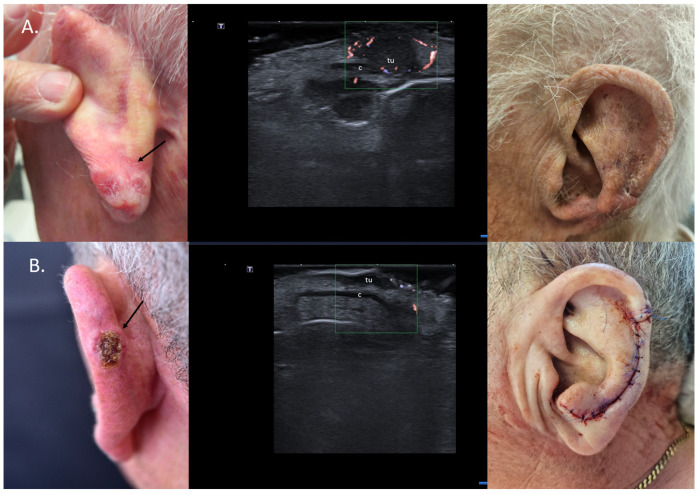
(Clinical aspect—left; HFUS-middle; postoperative aspect—right): (**A**) Erythematous plaque of the ear helix, as marked by “↑”, consistent with a solid BCC; HFUS and microvascular imaging showing a hypoechoic lesion, as marked by “tu“, with peripheral and basal vascularization with suspicion of cartilage infiltration, marked by “c”—longitudinal view; Clinical aspect 8 weeks after wedge excision; (**B**) Ulcerated nodule of the left helix, as marked by “↑”, consistent with an ulcerated BCC; HFUS and microvascular imaging show a hypoechoic lesion, as marked by “tu“, without any cartilage infiltration, marked by “c“—longitudinal view; Clinical aspect after performance of a chondrocutaneous flap.

For localized cartilage infiltration, wedge excisions at auricular or nasal level are easy to perform and enable defect reconstruction in one surgical step. In cases where cartilage infiltration is ruled out, chondrocutaneous flaps for the auricle or advancement/transposition flaps for the nasal area can be considered (Figure 7).

In cases of extensive cartilage infiltration, where curative resection is not possible, systemic therapies such as sonic hedgehog inhibitors for BCCs and PD1 antibodies for SCCs can be employed in a neoadjuvant approach, reducing the tumor size prior to surgery [2]. Particularly for the identification of cases with extensive cartilage infiltration that could benefit from systemic treatment prior to surgery, HFUS is an extremely reliable and precise imagistic method.

## 8. Preoperative Neuro-Vascular Mapping

Pre-interventional sonographic mapping of vascular structures is increasingly being used in aesthetics prior to hyaluronic acid injection in order to ensure safety against vascular occlusion caused by the filler [35].

In the aesthetic field, proper knowledge of facial anatomy is essential; however, there are considerable amounts of vascular anatomical variations, which cannot guarantee the safety of the injection procedure without proper vascular mapping in advance.

HFUS and color Doppler have proven to be essential tools for the prevention of vascular complications during aesthetic procedures [36].

**Figure 8:**
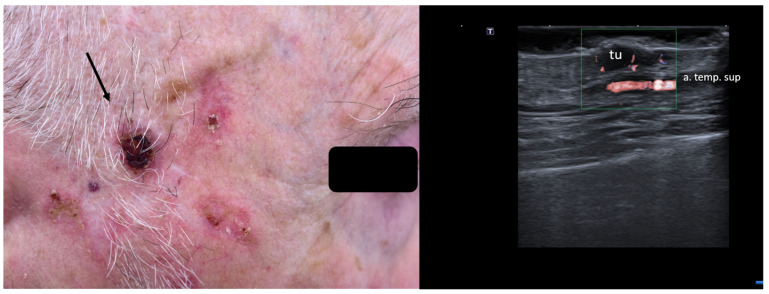
(Clinical aspect—left; HFUS—right): Hyperkeratotic plaque at right temporal area, as marked by “↑”, consistent with an SCC; HFUS and microvascular imaging show a hypoechoic, vascularized lesion, marked by “tu”, protruding above the frontal branch of the superficial temporal artery—longitudinal view.

When managing skin lesions, especially at the facial level, preoperative HFUS can hence aid in identifying arteries, veins, and even nerves prior to surgical intervention, as seen in Figure 8.

This is of paramount importance for surgeons, allowing a reduction in the risk of postoperative bleeding, nerve damage, or postoperative disfigurement. Further, this information is also essential in the preoperative setting when discussing the surgical steps, reconstructive options, expected outcomes, and potential complications [20].

## 9. Ex Vivo Tumor Margin Assessment

Ex vivo sonography for tumor-free margin assessment in skin cancer has been shown to be very precise and have a very good specificity [37,38].

Knowingly, depending on the tumor histological subtype, re-intervention after one surgical step can be necessary, especially in cases of irregular or ill-defined lesions.

Pasquali et al. reported on the use of HFUS for ex vivo tumor margin assessment, which would help increase the chances of a faster complete tumor excision [37].

Vilas-Sueriro et al. were also able to show in a prospective study on 65 patients with BCCs that ex vivo sonography of the tumor margins had a negative predictive value of 96%, indicating that US is a highly efficient tool in delimiting tumor involvement of surgical margins in BCCs and sparing surgical steps [38].

**Figure 9:**
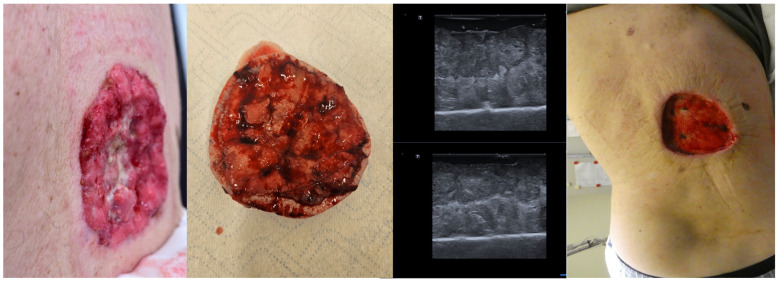
(Clinical aspect “in vivo” and “ex vivo”—left; HFUS—middle; postoperative aspect—right): Clinical aspect of an ulcerated plaque on the back, consistent with an ulcerated BCC, as seen “in vivo” and “ex vivo”; “ex vivo” HFUS delimitating the tumor margins in relation to the resection margin; Clinical aspect after complete tumor resection.

Even in cases with large tumors, as seen in Figure 9, ex vivo HFUS of the lesion can help identify tumor margins, and in cases of sonographic incomplete excision, an immediate re-intervention at that site is possible, increasing the chances of a full tumor resection in one surgical step and reducing the time needed for histological confirmation of tumor margins Figure 9.

## 10. Avoidance of Unnecessary Surgeries due to Patient Up-Staging

Preoperative HFUS of primary cutaneous tumors and, for certain entities, of the locoregional lymph nodes should become a routine in dermatology, as the examination might help to identify potential satellite or lymph nodal lesions which automatically upstage the patients in a higher TNM/AJCC stage, where the surgical approach might be fully canceled or adjusted [2]. It also prevents a delay in diagnosis and therapy initiation and spares patients, sometimes mutilating surgery.

**Figure 10:**
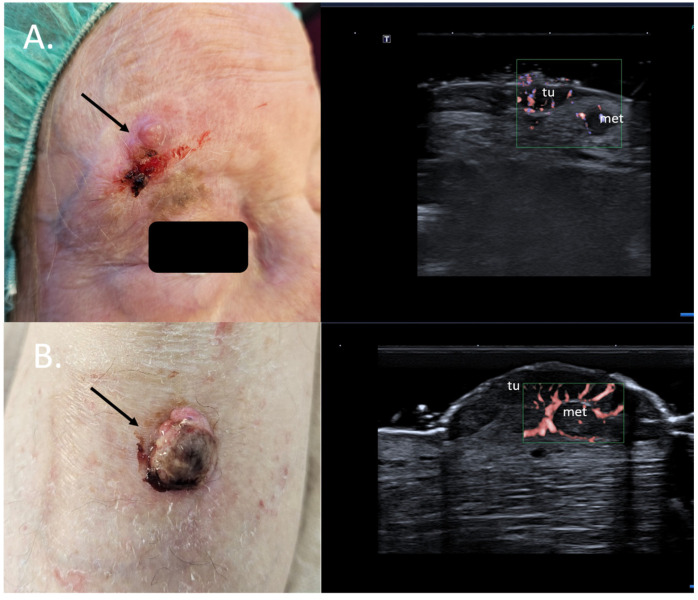
(Clinical aspect—left; HFUS-right): (**A**) Erythematous nodule at right frontal area, as marked by “↑”, consistent with an amelanotic MM; HFUS and microvascular imaging show a hypoechoic, intensely vascularized lesion as primary tumor, marked by “tu“ and a secondary dermal-hypodermal, hypoechoic, vascularized lesion situated laterally, suspicious of dermal metastasis, as marked by “met“, and confirmed by histology—transverse view; (**B**) Ulcerated nodule of the lower leg, as marked by “↑”, consistent with a nodular MM; HFUS and microvascular imaging identify a hypoechoic lesion beneath the primary tumor, marked by “tu“, with central and peripheral vascularization, consistent with a local satellite metastasis, as marked by “met“—transverse view.

Specifically, for MM, the preoperative sonographic assessment of the primary tumor can help identify dermal satellite lesions, unseen by the clinical examination and only identifiable on histology, immediately up-staging the patients to AJCC stage III. In such cases, a full body CT scan to rule out further metastatic disease should be immediately initiated, and the need for a sentinel node biopsy and a re-excision with a large safety margin becomes obsolete [39,40] Figure 10.

**Figure 11:**
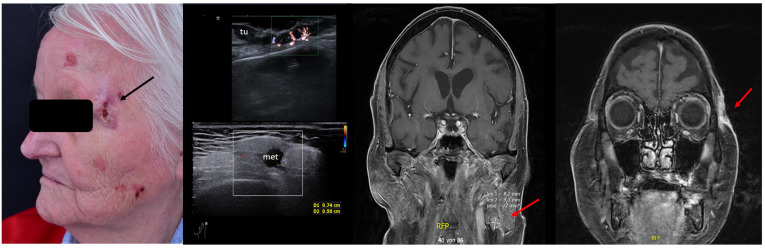
(Clinical aspect—left; HFUS—middle, MRI scan- right): Clinical aspect of an ulcerated plaque at the lateral orbital rim, as marked by “↑”, consistent with an ulcerated SCC; HFUS and microvascular imaging of the primary tumor and in-transit area showing no orbital rim infiltration but a suspicious hypoechoic lesion of the parotid gland, as marked by “met”, histologically confirmed as lymph node metastasis—longitudinal view; MRI showing the parotid lesion and the primary tumor with no bony invasion, as marked by “↑”.

For SCC, Merkel cell carcinoma, and MM, preoperative assessment of the locoregional lymph node stations prior to surgery can also help identify lymph nodal involvement at the time of diagnosis, which again alters the therapeutic management, avoiding unnecessary interventions in often frail patients. See Figure 11.

## 11. Intraoperative Guidance

**Figure 12:**
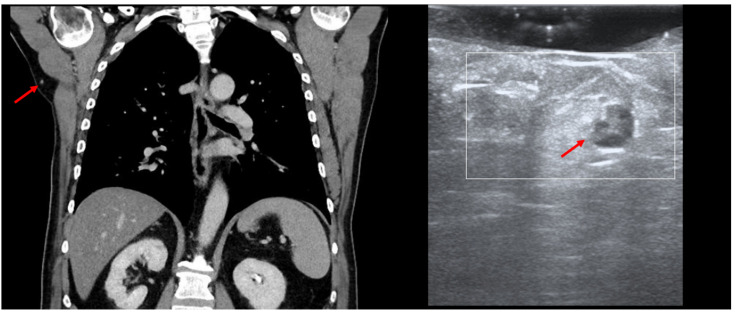
(PET-CT—left, US—right): Thoracic PET-CT showing a new lesion in the right axillary area in a MM patient, suspicious of metastatic disease, as marked by “↑”; Intraoperative HFUS showing a subcutaneous hypoechoic lesion, marked by “↑”, guiding the excision margins—transversal view.

HFUS can be employed in the intra-operative setting for the identification of certain occult lesions, as seen in Figure 12. 

In this MM patient, PET-CT showed a new subcutaneous lesion in the right axillary area, which we were able to detect and remove by performing intraoperative US, as the lesion only measured 7 mm. In cases of small/deep metastases, their marking can also be performed using a percutaneous guidewire [41]. The application of the guidewire usually occurs without anesthesia by directing the wire under US guidance toward the lesion. Once the lesion has been identified, the needle is removed in order to unfold the wire ends, and the wire is fixed at cutaneous level, enabling the surgeon to then remove the marked lesion [42]. However, similarly to the gamma probe for sentinel node biopsy, the transducer can be used in the intra-operative setting with the appropriate sterile covering to facilitate the identification of the target lesion without the need for a guidewire.

## 12. Conclusions

Skin cancer surgery can significantly benefit from the “in vivo” and “ex vivo” information provided by HFUS, which allows a very accurate and detailed assessment of the lesions and offers essential complementary data to clinical aspects, dermoscopy, and biopsy, which can alter the therapeutic approach. The ultrasonographic features identified by pre-therapeutical sonography and the histological tumor subtype can further aid in deciding upon the most effective treatment regimen.

Ultrasound examinations are rapid, cost-effective, painless, and non-invasive, emphasizing the potential implementation of sonography as a standard diagnostic and evaluation procedure in skin cancer patients. Not only does sonography eliminate the use of ionizing radiation, with significant safety benefits, but it also has generally lower costs in comparison to other imaging techniques, such as CT and MRI, which are not always and immediately available, often leading to a delay in therapy initiation. Furthermore, the pre- and post-therapeutical use of sonography in skin cancer patients could lead to significant cost reduction in the long term due to the early diagnosis and identification of local recurrences, which can be treated at an early stage while also improving the cosmetic appearance and functionality, which is essential for our patients. By providing a very good balance between resolution and penetration depth, sonography is a real-time technique that can surely improve our daily practice when diagnosing, treating, or monitoring skin cancer patients.

Pre-surgical HFUS should become a routine in skin cancer surgery for orienting the clinical diagnosis, improvement in the surgical planning, and identification of the best surgical/therapeutic approach, which could also lead to a decrease in healthcare, intervention-related costs (operating room time as a limited resource) and delay in therapy initiation.

Furthermore, HFUS is of significant value for the intra-operative detection and removal of small, non-palpable lesions suspicious of metastatic disease, helping to guide the surgeon to plan the operations precisely and lowering the risk of non-diagnostic resections.

## Data Availability

The data that support the findings of this study are available from the corresponding author, [D.C.], upon reasonable request.
